# Aqua­(2,2′-bipyridine-κ^2^ *N*,*N*′)(3,5-dinitro­benzoato-κ*O* ^1^)copper(II) tetra­hydro­furan monosolvate

**DOI:** 10.1107/S1600536810030436

**Published:** 2010-08-04

**Authors:** Norbani Abdullah, M. I. Mohamadin, A. P. Safwan, Edward R. T. Tiekink

**Affiliations:** a Department of Chemistry, University of Malaya, 50603 Kuala Lumpur, Malaysia

## Abstract

The title complex, [Cu(C_7_H_3_N_2_O_6_)_2_(C_10_H_8_N_2_)(H_2_O)]·C_4_H_8_O, features a penta­coordinate Cu^II^ atom bound by two monodentate carboxyl­ate ligands, a bidentate 2,2′-bipyridine mol­ecule [dihedral angle between pyridine rings = 5.0 (2)°] and a water mol­ecule. The resulting N_2_O_3_ donor set defines a distorted square-pyramidal geometry with the coordinated water mol­ecule in the apical position. In the crystal, the presence of O—H_w_⋯O_c_ (w = water and c = carbon­yl) hydrogen bonding leads to the formation of a supra­molecular chain propagating along the *c* axis, which associates into a double chain *via* C—H⋯ O and π–π contacts between pyridyl rings [centroid–centroid distance = 3.527 (3) Å]. The solvent mol­ecules, which are disordered over two orientations in a 0.678 (11):0.322 (11) ratio, occupy voids defined by the complex mol­ecules and are held in place *via* C—H⋯O inter­actions.

## Related literature

For background to the study of copper carboxyl­ates, see: Ozair *et al.* (2010 ▶). For the preparation, see: Fountain & Hatfield (1965 ▶). For additional geometric analysis, see: Addison *et al.* (1984 ▶).
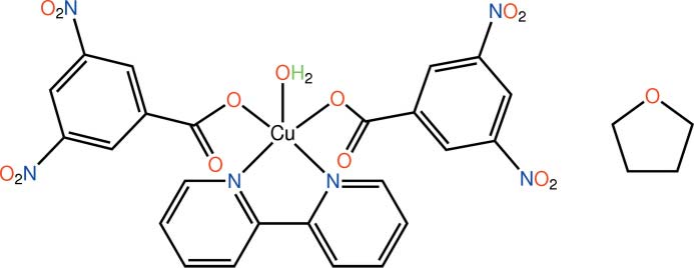


## Experimental

### 

#### Crystal data

[Cu(C_7_H_3_N_2_O_6_)_2_(C_10_H_8_N_2_)(H_2_O)]·C_4_H_8_O
*M* *_r_* = 732.08
Orthorhombic, 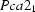

*a* = 19.6424 (7) Å
*b* = 23.2687 (8) Å
*c* = 6.5897 (2) Å
*V* = 3011.84 (17) Å^3^
*Z* = 4
Mo *K*α radiation
μ = 0.81 mm^−1^
*T* = 100 K
0.26 × 0.07 × 0.07 mm

#### Data collection

Bruker SMART APEX CCD diffractometer
Absorption correction: multi-scan (*SADABS*; Sheldrick, 1996 ▶) *T*_min_ = 0.865, *T*_max_ = 1.000
25602 measured reflections
6223 independent reflections
5759 reflections with *I* > 2σ(*I*)
*R*_int_ = 0.035

#### Refinement

*R*[*F* ^2^ > 2σ(*F* ^2^)] = 0.060
*wR*(*F* ^2^) = 0.134
*S* = 1.28
6223 reflections
444 parameters
14 restraints
H atoms treated by a mixture of independent and constrained refinement
Δρ_max_ = 0.50 e Å^−3^
Δρ_min_ = −0.50 e Å^−3^
Absolute structure: Flack (1983 ▶), 2809 Friedel pairs
Flack parameter: 0.02 (2)

### 

Data collection: *APEX2* (Bruker, 2009 ▶); cell refinement: *SAINT* (Bruker, 2009 ▶); data reduction: *SAINT*; program(s) used to solve structure: *SHELXS97* (Sheldrick, 2008 ▶); program(s) used to refine structure: *SHELXL97* (Sheldrick, 2008 ▶); molecular graphics: *ORTEP-3* (Farrugia, 1997 ▶) and *DIAMOND* (Brandenburg, 2006 ▶); software used to prepare material for publication: *publCIF* (Westrip, 2010 ▶).

## Supplementary Material

Crystal structure: contains datablocks global, I. DOI: 10.1107/S1600536810030436/hb5590sup1.cif

Structure factors: contains datablocks I. DOI: 10.1107/S1600536810030436/hb5590Isup2.hkl

Additional supplementary materials:  crystallographic information; 3D view; checkCIF report

## Figures and Tables

**Table 1:** Selected bond lengths (Å)

Cu—O7	1.951 (4)
Cu—O1	1.972 (4)
Cu—N5	2.007 (4)
Cu—N6	2.010 (4)
Cu—O1*W*	2.198 (4)

**Table 2:** Hydrogen-bond geometry (Å, °)

*D*—H⋯*A*	*D*—H	H⋯*A*	*D*⋯*A*	*D*—H⋯*A*
O1w—H1w⋯O2^i^	0.84 (5)	2.01 (6)	2.766 (6)	150 (7)
O1w—H2w⋯O8^i^	0.84 (5)	2.35 (7)	3.048 (6)	141 (6)
C18—H18⋯O8^ii^	0.95	2.17	3.060 (7)	155
C21—H21⋯O2^ii^	0.95	2.41	3.087 (7)	128
C15—H15⋯O9^i^	0.95	2.43	3.285 (7)	150
C1s—H1s2⋯O7	0.99	2.53	3.451 (10)	155
C3—H3⋯O1*s*	0.95	2.58	3.520 (8)	169
C2s—H2s2⋯O11	0.99	2.49	3.385 (11)	150
C5—H5⋯O4^iii^	0.95	2.58	3.360 (7)	140
C12—H12⋯O12^iii^	0.95	2.43	3.263 (7)	146
C16—H16⋯O12^iv^	0.95	2.47	3.234 (8)	138

Symmetry codes: (i) 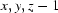; (ii) 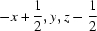; (iii) 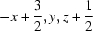; (iv) 
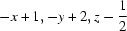
.

**Fig. 1.**
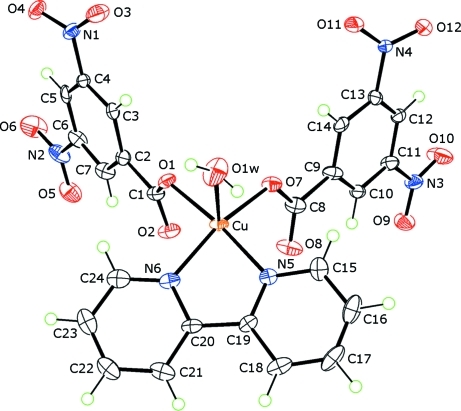
The molecular structure of (I) showing displacement ellipsoids at the 50% probability level. The disordered thf molecule is not illustrated.

**Fig. 2.**
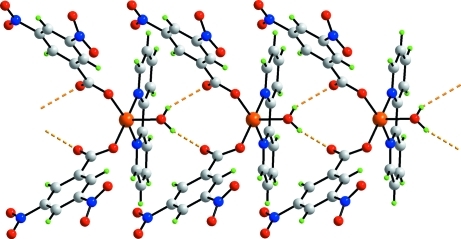
A portion of the supramolecular chain aligned along the c axis found in the crystal structure of (I) mediated by O–H···O hydrogen bonding (orange dashed lines). Colour code: Cu, orange; O, red; N, blue; C, grey; and H, green.

**Fig. 3.**
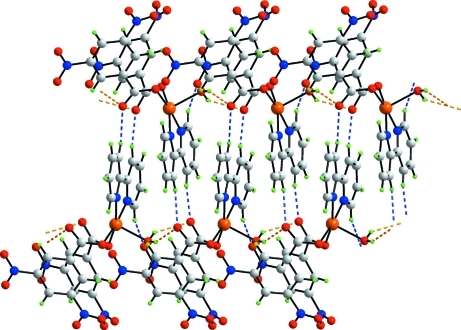
Double chain with helical topology in (I) mediated by O–H···O and C–H···O contacts shown as orange and blue dashed lines, respectively. Colour code: Cu, orange; O, red; N, blue; C, grey; and H, green.

**Fig. 4.**
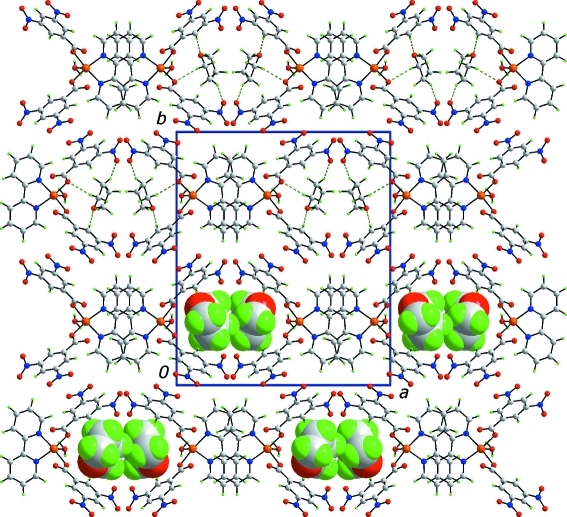
View of the unit-cell contents of (I) viewed in projection down the c axis. The double chains form layers in the ac plane which have large voids that are occupied by the solvent thf molecules; only the major component of the disordered molecules are shown. In the lower two layers, the solvent molecules are shown in space filling mode. In the upper two layers, the C–H···O interactions connecting the thf molecules to the layers are shown as green dashed lines. Colour code: Cu, orange; O, red; N, blue; C, grey; and H, green.

## supplementary crystallographic information

### Comment

The title complex solvate, (I), was characterized as part of on-going structural studies of copper carboxylates and their adducts (Ozair *et al.*, 2010).

The crystallographic asymmetric unit of (I) comprises a copper(II) complex and a solvent tetrahydrofuran (thf) molecule of crystallization in a 1:1 ratio. The molecular structure of the complex in (I) is illustrated in Fig. 1 and selected geometric parameters are collected in Table 1. The Cu atom is penta-coordinate, being coordinated by two O atoms derived from two monodentate carboxylate ligand, two N atoms of the chelating 2,2'-bipyridine ligand, and an O atom derived from the coordinated water molecule. The resulting N_2_O_3_ donor set defines a square pyramidal geometry as indicated by the value of τ = 0.16 which compares to τ = 0 for an ideal square pyramid and τ = 1.0 for an ideal trigonal bipyramid (Addison *et al.*, 1984). In this description, the coordinated water molecule occupies the apical position and each carboxylate-O atom is *trans* to a pyridine-N atom, Table 2. The four donor atoms defining the square plane have deviations from the least-squares plane through them of -0.089 (2), 0.090 (2), -0.097 (2), and 0.096 (2) Å for atoms O1, O7, N5, and N6, respectively; the r.m.s. deviation for the four atoms is 0.093 Å. The Cu atom lies 0.176 (2) Å out of the square plane in the direction of the O1w atom. Distortions from the ideal geometry are due to the restricted bite distance of the 2,2'-bipyridine ligand [N5–Cu–N6 = 79.95 (18) °] and to the relatively close approach of the carbonyl-O2, O8 atoms. However, the Cu···O2, O4 separations of 2.942 (4) and 3.007 (4) Å, respectively, are not considered to represent significant bonding interactions. Under these circumstances, the disparity in the *C*–O_carboxylate_ and *C*–O_carbonyl_ bond distances, Table 1, is not as great as might be anticipated for formal *C*–O_carboxylate_ and C═O_carbonyl_ bonds. This is due to i) the weak interaction formed by the carbonyl-O atoms with the Cu atom, and ii) the pivotal role the carbonyl-O atoms play in the supramolecular association operating in the crystal structure (see below). Each of the carbonyl-O2,O8 atoms lies to the same side of the square plane around the Cu atom and in the opposite direction to the coordinated water molecule. The dihedral angle formed between the two carboxylate aromatic rings is 82.1 (2) °, indicating that they are almost orthogonal to each other. Within the carboxylate ligands, each carboxylate group is effectively co-planar with the aromatic ring to which it is bound, with the C1–O1,O2 carboxylate having the greater twist as seen in the O1–C1–C2–C3 torsion angle of 10.0 (7) °. By contrast, one nitro group in each carboxylate ligand, *i.e*. containing N1 and N4, is significantly twisted out of the plane of the aromatic ring to which it is connected [the O3–N1–C4–C3 and O11–N4–C13–C12 torsion angles are -162.7 (5) and 157.4 (5) °, respectively]. The chelating 2,2'-bipyridine ligand is almost planar with the dihedral angle between the two pyridine rings being 5.0 (2) °; the small twist in the molecule is seen in the N5–C19–C20–N6 torsion angle of -2.6 (7) °.

The most prominent interactions operating in the crystal structure of (I) are O–H···O contacts occurring between the hydrogen atoms of the coordinated water molecule and the carbonyl-O atoms of a translationally related molecule; Table 2. As illustrated in Fig. 2, the water-bound hydrogen atoms effectively form a bridge between the adjacent carbonyl atoms resulting in a ten-membered {···HOH···OCOCuOCO} synthon. The result of this hydrogen bonding is the formation of a supramolecular chain along the *c* axis. Each supramolecular chain is connected into a double chain along *c* with helical topology *via* C–H···O contacts whereby two bipyridine-H atoms form interactions with a carbonyl-O of the second chain, and a third bipyridine-H atom forms a C–H···O contact with a nitro-O within the chain, Fig. 3 and Table 2. This arrangement brings into close proximity the 2,2'-bipyridine molecules which interdigitate, Fig. 3, allowing for the formation of π–π interactions [ring centroid(N6,C20–C24)···ring centroid(N6,C20–C24)^i^ = 3.527 (3) Å for i: -*x* + 1/2, *y*, *z* - 1/2]. The double chains pack in the *ac* plane to form layers that stack along the *b* axis, Fig. 4. Within each layer, there are voids and these are occupied by the solvent thf molecules which are held in place by C–H···O interactions, Table 2 and Fig. 4. Interactions between layers are primarily of the type C–H···O as detailed in Table 2.

### Experimental

Copper(II) acetate monohydrate (Merck; 1.995 g, 0.01 mol) and 3,5-dinitrobenzoic acid (Merck, 4.24 g, 0.02 mol) were reacted in an 1:2 molar ratio hot ethanol (60 ml) for 30 minutes following a literature precedent (Fountain & Hatfield, 1965). The resulting blue powder, [Cu_2_(3,5-(NO_2_)_2_C_6_H_3_COO)_4_], was isolated in 23% yield and reacted with 2,2'-bipyridine (mole ratio = 1:1) in THF (15 ml) at room temperature. Blue-green prisms of (I) formed when the solvent was allowed to slowly evaporate off at room temperature after 2 days.

### Refinement

Carbon-bound H-atoms were placed in calculated positions (C—H 0.95 to 0.99 Å) and were included in the refinement in the riding model approximation, with *U*_iso_(H) set to 1.2*U*_equiv_(C). The water-bound H-atoms were located in a difference Fourier map but were were refined with a distance restraints of O–H = 0.84±0.01 Å and H···H = 1.39±0.05 Å, and with *U*_iso_(H) = 1.5*U*_eq_(O). The complex was found to crystallize as a 1:1 tetrahydrofuran (thf) solvate. The solvent thf molecule was found to be disordered and resolved over two distinct orientations *via* fractional refinement. The major component of the disorder, with a site occupancy factor = 0.678 (11), was refined with anisotropic displacement parameters but, the non-hydrogen atoms comprising the minor component were refined isotropically. Finally, the distance restraints C–O = 1.40±0.01 Å and C—C = 1.50±0.01 Å were applied to the disordered atoms. While there is an indication of pseudo C-centring (excluding the disordered atoms), there are no counterparts for atoms O3 and O11.

### Crystal data

**Table:**

[Cu(C_7_H_3_N_2_O_6_)_2_(C_10_H_8_N_2_)(H_2_O)]·C_4_H_8_O	*F*(000) = 1500
*M**_r_* = 732.08	*D*_x_ = 1.614 Mg m^−^^3^
Orthorhombic, *P**c**a*2_1_	Mo *K*α radiation, λ = 0.71073 Å
Hall symbol: P 2c -2ac	Cell parameters from 7872 reflections
*a* = 19.6424 (7) Å	θ = 2.6–28.0°
*b* = 23.2687 (8) Å	µ = 0.81 mm^−^^1^
*c* = 6.5897 (2) Å	*T* = 100 K
*V* = 3011.84 (17) Å^3^	Prism, green
*Z* = 4	0.26 × 0.07 × 0.07 mm

### Data collection

**Table:**

Bruker SMART APEX CCD diffractometer	6223 independent reflections
Radiation source: fine-focus sealed tube	5759 reflections with *I* > 2σ(*I*)
graphite	*R*_int_ = 0.035
ω scans	θ_max_ = 26.5°, θ_min_ = 0.9°
Absorption correction: multi-scan (*SADABS*; Sheldrick, 1996)	*h* = −24→23
*T*_min_ = 0.865, *T*_max_ = 1.000	*k* = −29→29
25602 measured reflections	*l* = −8→8

### Refinement

**Table:**

Refinement on *F*^2^	Secondary atom site location: difference Fourier map
Least-squares matrix: full	Hydrogen site location: inferred from neighbouring sites
*R*[*F*^2^ > 2σ(*F*^2^)] = 0.060	H atoms treated by a mixture of independent and constrained refinement
*wR*(*F*^2^) = 0.134	*w* = 1/[σ^2^(*F*_o_^2^) + (0.0125*P*)^2^ + 10.1609*P*] where *P* = (*F*_o_^2^ + 2*F*_c_^2^)/3
*S* = 1.28	(Δ/σ)_max_ < 0.001
6223 reflections	Δρ_max_ = 0.50 e Å^−^^3^
444 parameters	Δρ_min_ = −0.50 e Å^−^^3^
14 restraints	Absolute structure: Flack (1983), 2809 Friedel pairs
Primary atom site location: structure-invariant direct methods	Flack parameter: 0.02 (2)

### Special details

**Table:**

Geometry. All s.u.'s (except the s.u. in the dihedral angle between two l.s. planes) are estimated using the full covariance matrix. The cell s.u.'s are taken into account individually in the estimation of s.u.'s in distances, angles and torsion angles; correlations between s.u.'s in cell parameters are only used when they are defined by crystal symmetry. An approximate (isotropic) treatment of cell s.u.'s is used for estimating s.u.'s involving l.s. planes.
Refinement. Refinement of *F*^2^ against ALL reflections. The weighted *R*-factor *wR* and goodness of fit *S* are based on *F*^2^, conventional *R*-factors *R* are based on *F*, with *F* set to zero for negative *F*^2^. The threshold expression of *F*^2^ > 2σ(*F*^2^) is used only for calculating *R*-factors(gt) *etc*. and is not relevant to the choice of reflections for refinement. *R*-factors based on *F*^2^ are statistically about twice as large as those based on *F*, and *R*- factors based on ALL data will be even larger.

### Fractional atomic coordinates and isotropic or equivalent isotropic displacement parameters (Å^2^)

**Table:**

	*x*	*y*	*z*	*U*_iso_*/*U*_eq_	Occ. (<1)
Cu	0.42505 (3)	0.74892 (3)	0.81422 (13)	0.01710 (13)	
O1	0.48462 (18)	0.68415 (15)	0.8897 (5)	0.0203 (8)	
O2	0.43340 (18)	0.68207 (18)	1.1923 (6)	0.0276 (9)	
O3	0.7080 (2)	0.59152 (17)	0.8280 (8)	0.0349 (10)	
O4	0.7241 (2)	0.51293 (17)	0.9989 (6)	0.0277 (9)	
O5	0.5108 (2)	0.5410 (2)	1.6840 (6)	0.0362 (11)	
O6	0.6025 (2)	0.4906 (2)	1.6345 (8)	0.0475 (13)	
O7	0.49031 (18)	0.80342 (17)	0.9272 (6)	0.0223 (8)	
O8	0.41911 (19)	0.8246 (2)	1.1836 (6)	0.0313 (10)	
O9	0.4959 (2)	0.95628 (19)	1.7149 (7)	0.0326 (10)	
O10	0.5952 (2)	0.9953 (2)	1.6972 (8)	0.0447 (13)	
O11	0.7198 (2)	0.88618 (18)	0.9376 (7)	0.0344 (10)	
O12	0.73480 (19)	0.96655 (16)	1.0991 (7)	0.0300 (10)	
O1W	0.4763 (3)	0.7479 (2)	0.5176 (6)	0.0417 (11)	
H2W	0.450 (3)	0.774 (2)	0.478 (11)	0.063*	
H1W	0.478 (4)	0.723 (2)	0.426 (8)	0.063*	
N1	0.6927 (2)	0.5575 (2)	0.9613 (7)	0.0232 (10)	
N2	0.5602 (3)	0.5278 (2)	1.5819 (8)	0.0303 (11)	
N3	0.5503 (2)	0.9650 (2)	1.6327 (7)	0.0240 (10)	
N4	0.7016 (2)	0.92304 (18)	1.0584 (8)	0.0209 (10)	
N5	0.3550 (2)	0.80942 (19)	0.7545 (6)	0.0169 (9)	
N6	0.3428 (2)	0.6991 (2)	0.7701 (6)	0.0198 (10)	
C1	0.4777 (2)	0.6669 (2)	1.0699 (8)	0.0192 (11)	
C2	0.5301 (3)	0.6231 (2)	1.1368 (8)	0.0180 (11)	
C3	0.5867 (3)	0.6110 (2)	1.0177 (8)	0.0173 (10)	
H3	0.5934	0.6296	0.8911	0.021*	
C4	0.6333 (2)	0.5706 (2)	1.0894 (8)	0.0175 (10)	
C5	0.6267 (3)	0.5431 (2)	1.2735 (8)	0.0199 (12)	
H5	0.6593	0.5159	1.3196	0.024*	
C6	0.5703 (3)	0.5571 (2)	1.3865 (8)	0.0243 (12)	
C7	0.5218 (3)	0.5960 (2)	1.3246 (10)	0.0248 (11)	
H7	0.4836	0.6043	1.4077	0.030*	
C8	0.4735 (3)	0.8296 (2)	1.0928 (8)	0.0225 (11)	
C9	0.5270 (3)	0.8696 (2)	1.1773 (8)	0.0210 (11)	
C10	0.5142 (3)	0.8988 (2)	1.3576 (8)	0.0181 (11)	
H10	0.4721	0.8937	1.4260	0.022*	
C11	0.5628 (3)	0.9350 (2)	1.4367 (9)	0.0219 (11)	
C12	0.6256 (3)	0.9422 (2)	1.3450 (8)	0.0178 (11)	
H12	0.6599	0.9654	1.4048	0.021*	
C13	0.6362 (3)	0.9144 (2)	1.1637 (9)	0.0205 (11)	
C14	0.5891 (2)	0.8771 (2)	1.0798 (8)	0.0178 (10)	
H14	0.5989	0.8570	0.9579	0.021*	
C15	0.3682 (3)	0.8653 (2)	0.7354 (8)	0.0257 (12)	
H15	0.4139	0.8784	0.7420	0.031*	
C16	0.3158 (4)	0.9047 (3)	0.7059 (9)	0.0345 (15)	
H16	0.3260	0.9444	0.6915	0.041*	
C17	0.2505 (4)	0.8867 (3)	0.6977 (9)	0.0320 (14)	
H17	0.2147	0.9136	0.6792	0.038*	
C18	0.2362 (3)	0.8287 (3)	0.7166 (8)	0.0252 (12)	
H18	0.1906	0.8152	0.7129	0.030*	
C19	0.2910 (3)	0.7905 (2)	0.7414 (7)	0.0161 (10)	
C20	0.2837 (3)	0.7281 (2)	0.7550 (7)	0.0187 (11)	
C21	0.2213 (3)	0.6990 (3)	0.7491 (8)	0.0256 (12)	
H21	0.1797	0.7197	0.7411	0.031*	
C22	0.2212 (3)	0.6388 (3)	0.7554 (8)	0.0289 (13)	
H22	0.1795	0.6182	0.7526	0.035*	
C23	0.2816 (3)	0.6102 (3)	0.7655 (8)	0.0292 (13)	
H23	0.2826	0.5694	0.7675	0.035*	
C24	0.3417 (3)	0.6415 (3)	0.7727 (7)	0.0259 (13)	
H24	0.3836	0.6213	0.7799	0.031*	
O1S	0.6126 (3)	0.6981 (3)	0.5879 (10)	0.0375 (19)*	0.678 (11)
C1S	0.6344 (5)	0.7402 (3)	0.7275 (13)	0.029 (2)*	0.678 (11)
H1S1	0.6734	0.7259	0.8083	0.035*	0.678 (11)
H1S2	0.5970	0.7507	0.8211	0.035*	0.678 (11)
C2S	0.6551 (5)	0.7910 (4)	0.6005 (15)	0.039 (2)*	0.678 (11)
H2S1	0.6153	0.8155	0.5682	0.047*	0.678 (11)
H2S2	0.6898	0.8145	0.6711	0.047*	0.678 (11)
C4S	0.6573 (6)	0.7039 (5)	0.4224 (18)	0.059 (3)*	0.678 (11)
H4S1	0.6331	0.6944	0.2950	0.070*	0.678 (11)
H4S2	0.6958	0.6767	0.4377	0.070*	0.678 (11)
C3S	0.6839 (6)	0.7640 (4)	0.4117 (17)	0.053 (3)*	0.678 (11)
H3S1	0.6675	0.7839	0.2882	0.064*	0.678 (11)
H3S2	0.7343	0.7647	0.4132	0.064*	0.678 (11)
O2S	0.6819 (9)	0.7085 (7)	0.296 (3)	0.070 (6)*	0.322 (11)
C5S	0.6454 (10)	0.7603 (7)	0.299 (3)	0.041 (5)*	0.322 (11)
H5S1	0.5993	0.7537	0.2429	0.049*	0.322 (11)
H5S2	0.6686	0.7888	0.2115	0.049*	0.322 (11)
C8S	0.6827 (10)	0.6886 (8)	0.496 (2)	0.032 (5)*	0.322 (11)
H8S1	0.7301	0.6874	0.5465	0.038*	0.322 (11)
H8S2	0.6638	0.6492	0.5013	0.038*	0.322 (11)
C7S	0.6406 (14)	0.7281 (9)	0.626 (4)	0.058 (7)*	0.322 (11)
H7S1	0.5940	0.7128	0.6442	0.069*	0.322 (11)
H7S2	0.6618	0.7333	0.7612	0.069*	0.322 (11)
C6S	0.6395 (15)	0.7838 (9)	0.511 (3)	0.058 (7)*	0.322 (11)
H6S1	0.6785	0.8088	0.5462	0.070*	0.322 (11)
H6S2	0.5963	0.8051	0.5310	0.070*	0.322 (11)

### Atomic displacement parameters (Å^2^)

**Table:**

	*U*^11^	*U*^22^	*U*^33^	*U*^12^	*U*^13^	*U*^23^
Cu	0.0121 (2)	0.0260 (3)	0.0132 (2)	0.0013 (3)	−0.0011 (3)	−0.0022 (3)
O1	0.0154 (18)	0.0217 (19)	0.024 (2)	0.0063 (15)	−0.0003 (14)	−0.0014 (15)
O2	0.0168 (19)	0.040 (2)	0.026 (2)	0.0046 (17)	−0.0002 (16)	−0.0089 (18)
O3	0.031 (2)	0.035 (2)	0.039 (2)	0.0110 (17)	0.013 (2)	0.012 (2)
O4	0.027 (2)	0.022 (2)	0.034 (2)	0.0091 (17)	−0.0020 (18)	−0.0005 (17)
O5	0.039 (2)	0.043 (3)	0.026 (2)	−0.012 (2)	0.007 (2)	0.0052 (19)
O6	0.037 (3)	0.054 (3)	0.051 (3)	0.001 (2)	−0.006 (2)	0.032 (3)
O7	0.0161 (18)	0.030 (2)	0.021 (2)	0.0004 (16)	−0.0012 (15)	−0.0019 (17)
O8	0.0181 (19)	0.054 (3)	0.022 (2)	−0.0079 (19)	0.0015 (17)	−0.0053 (19)
O9	0.029 (2)	0.037 (2)	0.031 (2)	−0.0051 (19)	0.0120 (19)	−0.0090 (19)
O10	0.024 (2)	0.059 (3)	0.051 (3)	−0.013 (2)	0.009 (2)	−0.029 (3)
O11	0.024 (2)	0.029 (2)	0.050 (3)	−0.0030 (18)	0.016 (2)	−0.010 (2)
O12	0.023 (2)	0.0222 (19)	0.045 (3)	−0.0030 (16)	0.0079 (19)	−0.0043 (19)
O1W	0.063 (3)	0.041 (3)	0.021 (2)	0.009 (3)	0.016 (2)	−0.002 (2)
N1	0.019 (2)	0.023 (2)	0.027 (2)	0.0073 (19)	−0.0023 (19)	−0.003 (2)
N2	0.028 (3)	0.039 (3)	0.024 (3)	−0.011 (2)	0.000 (2)	0.007 (2)
N3	0.019 (2)	0.028 (3)	0.025 (2)	0.001 (2)	0.004 (2)	−0.006 (2)
N4	0.0121 (19)	0.016 (2)	0.035 (3)	0.0009 (17)	0.0024 (19)	−0.001 (2)
N5	0.018 (2)	0.022 (2)	0.010 (2)	0.0017 (17)	−0.0014 (16)	0.0017 (17)
N6	0.017 (2)	0.030 (2)	0.012 (2)	0.0038 (18)	−0.0020 (17)	0.0009 (17)
C1	0.014 (2)	0.026 (3)	0.017 (3)	−0.001 (2)	−0.001 (2)	−0.009 (2)
C2	0.019 (2)	0.019 (3)	0.017 (3)	−0.002 (2)	0.001 (2)	−0.007 (2)
C3	0.019 (2)	0.016 (2)	0.017 (2)	−0.004 (2)	−0.004 (2)	−0.009 (2)
C4	0.014 (2)	0.016 (2)	0.022 (3)	−0.0012 (19)	0.000 (2)	−0.007 (2)
C5	0.023 (3)	0.011 (2)	0.026 (3)	−0.0038 (19)	−0.008 (2)	−0.004 (2)
C6	0.024 (3)	0.030 (3)	0.019 (2)	−0.008 (2)	−0.003 (2)	0.005 (2)
C7	0.022 (2)	0.034 (3)	0.018 (3)	−0.005 (2)	0.000 (3)	−0.003 (3)
C8	0.018 (3)	0.033 (3)	0.017 (3)	−0.001 (2)	−0.005 (2)	0.002 (2)
C9	0.013 (2)	0.032 (3)	0.018 (3)	0.001 (2)	−0.002 (2)	0.010 (2)
C10	0.014 (2)	0.020 (2)	0.020 (3)	0.000 (2)	0.001 (2)	0.001 (2)
C11	0.016 (2)	0.027 (3)	0.023 (3)	0.002 (2)	−0.001 (2)	−0.004 (2)
C12	0.015 (2)	0.017 (2)	0.021 (3)	0.0021 (18)	−0.001 (2)	−0.001 (2)
C13	0.016 (2)	0.016 (2)	0.030 (3)	0.001 (2)	0.000 (2)	0.011 (2)
C14	0.018 (2)	0.017 (2)	0.018 (2)	0.006 (2)	−0.002 (2)	0.005 (2)
C15	0.036 (3)	0.023 (3)	0.018 (2)	−0.002 (2)	−0.006 (2)	−0.001 (2)
C16	0.058 (4)	0.023 (3)	0.022 (3)	0.013 (3)	−0.009 (3)	0.004 (2)
C17	0.045 (4)	0.034 (3)	0.017 (3)	0.024 (3)	−0.010 (3)	−0.003 (2)
C18	0.022 (3)	0.042 (3)	0.011 (2)	0.012 (2)	−0.005 (2)	−0.005 (2)
C19	0.020 (3)	0.023 (3)	0.006 (2)	0.006 (2)	−0.0025 (19)	−0.003 (2)
C20	0.014 (2)	0.032 (3)	0.010 (2)	0.002 (2)	0.0001 (18)	−0.008 (2)
C21	0.021 (3)	0.047 (3)	0.010 (2)	−0.008 (3)	−0.001 (2)	−0.009 (2)
C22	0.032 (3)	0.040 (3)	0.014 (3)	−0.015 (3)	−0.004 (2)	−0.003 (2)
C23	0.045 (3)	0.029 (3)	0.014 (3)	−0.013 (3)	−0.003 (2)	0.000 (2)
C24	0.024 (3)	0.040 (3)	0.013 (3)	0.000 (2)	0.000 (2)	−0.005 (2)

### Geometric parameters (Å, °)

**Table:**

Cu—O7	1.951 (4)	C13—C14	1.384 (7)
Cu—O1	1.972 (4)	C14—H14	0.9500
Cu—N5	2.007 (4)	C15—C16	1.392 (8)
Cu—N6	2.010 (4)	C15—H15	0.9500
Cu—O1W	2.198 (4)	C16—C17	1.351 (10)
C1—O1	1.261 (6)	C16—H16	0.9500
C1—O2	1.238 (6)	C17—C18	1.382 (8)
O3—N1	1.220 (6)	C17—H17	0.9500
O4—N1	1.231 (6)	C18—C19	1.406 (7)
O5—N2	1.221 (7)	C18—H18	0.9500
O6—N2	1.248 (7)	C19—C20	1.461 (8)
C8—O7	1.293 (7)	C20—C21	1.402 (7)
C8—O8	1.230 (6)	C21—C22	1.400 (8)
O9—N3	1.214 (6)	C21—H21	0.9500
O10—N3	1.207 (6)	C22—C23	1.363 (9)
O11—N4	1.224 (6)	C22—H22	0.9500
O12—N4	1.234 (6)	C23—C24	1.387 (8)
O1W—H2W	0.84 (5)	C23—H23	0.9500
O1W—H1W	0.84 (5)	C24—H24	0.9500
N1—C4	1.473 (6)	O1S—C4S	1.406 (8)
N2—C6	1.470 (7)	O1S—C1S	1.410 (8)
N3—C11	1.489 (7)	C1S—C2S	1.505 (8)
N4—C13	1.473 (7)	C1S—H1S1	0.9900
N5—C15	1.331 (7)	C1S—H1S2	0.9900
N5—C19	1.336 (6)	C2S—C3S	1.504 (9)
N6—C24	1.340 (7)	C2S—H2S1	0.9900
N6—C20	1.346 (6)	C2S—H2S2	0.9900
C1—C2	1.514 (7)	C4S—C3S	1.496 (9)
C2—C3	1.389 (7)	C4S—H4S1	0.9900
C2—C7	1.399 (8)	C4S—H4S2	0.9900
C3—C4	1.394 (7)	C3S—H3S1	0.9900
C3—H3	0.9500	C3S—H3S2	0.9900
C4—C5	1.378 (7)	O2S—C8S	1.397 (10)
C5—C6	1.374 (8)	O2S—C5S	1.401 (10)
C5—H5	0.9500	C5S—C6S	1.503 (10)
C6—C7	1.376 (8)	C5S—H5S1	0.9900
C7—H7	0.9500	C5S—H5S2	0.9900
C8—C9	1.511 (7)	C8S—C7S	1.506 (10)
C9—C10	1.392 (7)	C8S—H8S1	0.9900
C9—C14	1.388 (7)	C8S—H8S2	0.9900
C10—C11	1.375 (7)	C7S—C6S	1.503 (10)
C10—H10	0.9500	C7S—H7S1	0.9900
C11—C12	1.383 (7)	C7S—H7S2	0.9900
C12—C13	1.376 (8)	C6S—H6S1	0.9900
C12—H12	0.9500	C6S—H6S2	0.9900
			
O7—Cu—O1	90.62 (16)	C17—C16—C15	120.2 (6)
O7—Cu—N5	93.96 (16)	C17—C16—H16	119.9
O1—Cu—N5	172.85 (17)	C15—C16—H16	119.9
O7—Cu—N6	163.43 (16)	C16—C17—C18	119.5 (5)
O1—Cu—N6	94.15 (17)	C16—C17—H17	120.3
N5—Cu—N6	79.95 (18)	C18—C17—H17	120.3
O7—Cu—O1W	92.59 (18)	C17—C18—C19	118.2 (5)
O1—Cu—O1W	86.83 (17)	C17—C18—H18	120.9
N5—Cu—O1W	98.43 (18)	C19—C18—H18	120.9
N6—Cu—O1W	103.51 (19)	N5—C19—C18	121.3 (5)
C1—O1—Cu	114.6 (3)	N5—C19—C20	114.5 (4)
C8—O7—Cu	117.4 (3)	C18—C19—C20	124.2 (5)
Cu—O1W—H2W	89 (6)	N6—C20—C21	120.9 (5)
Cu—O1W—H1W	132 (6)	N6—C20—C19	114.8 (5)
H2W—O1W—H1W	108 (5)	C21—C20—C19	124.3 (5)
O3—N1—O4	124.7 (5)	C22—C21—C20	118.9 (5)
O3—N1—C4	118.2 (4)	C22—C21—H21	120.5
O4—N1—C4	117.1 (5)	C20—C21—H21	120.5
O5—N2—O6	123.4 (5)	C23—C22—C21	119.3 (5)
O5—N2—C6	118.2 (5)	C23—C22—H22	120.4
O6—N2—C6	118.4 (5)	C21—C22—H22	120.4
O10—N3—O9	125.7 (5)	C22—C23—C24	119.0 (5)
O10—N3—C11	117.3 (4)	C22—C23—H23	120.5
O9—N3—C11	117.0 (5)	C24—C23—H23	120.5
O11—N4—O12	124.2 (4)	N6—C24—C23	122.6 (5)
O11—N4—C13	117.7 (4)	N6—C24—H24	118.7
O12—N4—C13	118.1 (4)	C23—C24—H24	118.7
C15—N5—C19	119.9 (5)	C4S—O1S—C1S	104.5 (8)
C15—N5—Cu	124.8 (4)	O1S—C1S—C2S	105.4 (7)
C19—N5—Cu	115.2 (3)	O1S—C1S—H1S1	110.7
C24—N6—C20	119.2 (5)	C2S—C1S—H1S1	110.7
C24—N6—Cu	126.0 (4)	O1S—C1S—H1S2	110.7
C20—N6—Cu	114.5 (4)	C2S—C1S—H1S2	110.7
O2—C1—O1	126.8 (5)	H1S1—C1S—H1S2	108.8
O2—C1—C2	118.7 (5)	C3S—C2S—C1S	103.5 (8)
O1—C1—C2	114.5 (4)	C3S—C2S—H2S1	111.1
C3—C2—C7	120.0 (5)	C1S—C2S—H2S1	111.1
C3—C2—C1	121.1 (5)	C3S—C2S—H2S2	111.1
C7—C2—C1	118.9 (5)	C1S—C2S—H2S2	111.1
C2—C3—C4	118.1 (5)	H2S1—C2S—H2S2	109.0
C2—C3—H3	120.9	O1S—C4S—C3S	110.2 (9)
C4—C3—H3	120.9	O1S—C4S—H4S1	109.6
C5—C4—C3	123.3 (5)	C3S—C4S—H4S1	109.6
C5—C4—N1	118.9 (5)	O1S—C4S—H4S2	109.6
C3—C4—N1	117.8 (5)	C3S—C4S—H4S2	109.6
C6—C5—C4	116.3 (5)	H4S1—C4S—H4S2	108.1
C6—C5—H5	121.9	C4S—C3S—C2S	102.7 (8)
C4—C5—H5	121.9	C4S—C3S—H3S1	111.2
C7—C6—C5	123.6 (5)	C2S—C3S—H3S1	111.2
C7—C6—N2	118.2 (5)	C4S—C3S—H3S2	111.2
C5—C6—N2	118.2 (5)	C2S—C3S—H3S2	111.2
C6—C7—C2	118.7 (5)	H3S1—C3S—H3S2	109.1
C6—C7—H7	120.7	C8S—O2S—C5S	106.1 (17)
C2—C7—H7	120.7	O2S—C5S—C6S	111.5 (18)
O8—C8—O7	126.0 (5)	O2S—C5S—H5S1	109.3
O8—C8—C9	119.0 (5)	C6S—C5S—H5S1	109.3
O7—C8—C9	115.1 (5)	O2S—C5S—H5S2	109.3
C10—C9—C14	119.5 (5)	C6S—C5S—H5S2	109.3
C10—C9—C8	119.3 (5)	H5S1—C5S—H5S2	108.0
C14—C9—C8	121.1 (5)	O2S—C8S—C7S	109.3 (17)
C11—C10—C9	119.8 (5)	O2S—C8S—H8S1	109.8
C11—C10—H10	120.1	C7S—C8S—H8S1	109.8
C9—C10—H10	120.1	O2S—C8S—H8S2	109.8
C10—C11—C12	121.8 (5)	C7S—C8S—H8S2	109.8
C10—C11—N3	120.0 (5)	H8S1—C8S—H8S2	108.3
C12—C11—N3	118.0 (5)	C6S—C7S—C8S	104.2 (18)
C13—C12—C11	117.2 (5)	C6S—C7S—H7S1	110.9
C13—C12—H12	121.4	C8S—C7S—H7S1	110.9
C11—C12—H12	121.4	C6S—C7S—H7S2	110.9
C12—C13—C14	122.7 (5)	C8S—C7S—H7S2	110.9
C12—C13—N4	118.5 (5)	H7S1—C7S—H7S2	108.9
C14—C13—N4	118.8 (5)	C7S—C6S—C5S	98.9 (18)
C13—C14—C9	118.7 (5)	C7S—C6S—H6S1	112.0
C13—C14—H14	120.6	C5S—C6S—H6S1	112.0
C9—C14—H14	120.6	C7S—C6S—H6S2	112.0
N5—C15—C16	120.9 (6)	C5S—C6S—H6S2	112.0
N5—C15—H15	119.6	H6S1—C6S—H6S2	109.7
C16—C15—H15	119.6		
			
O7—Cu—O1—C1	84.5 (4)	C14—C9—C10—C11	0.5 (7)
N5—Cu—O1—C1	−45.3 (15)	C8—C9—C10—C11	179.5 (5)
N6—Cu—O1—C1	−79.6 (4)	C9—C10—C11—C12	−2.0 (8)
O1W—Cu—O1—C1	177.1 (4)	C9—C10—C11—N3	−178.2 (5)
O1—Cu—O7—C8	−109.3 (4)	O10—N3—C11—C10	178.4 (5)
N5—Cu—O7—C8	65.2 (4)	O9—N3—C11—C10	−0.7 (8)
N6—Cu—O7—C8	−2.4 (8)	O10—N3—C11—C12	2.1 (8)
O1W—Cu—O7—C8	163.8 (4)	O9—N3—C11—C12	−177.0 (5)
O7—Cu—N5—C15	20.9 (4)	C10—C11—C12—C13	4.0 (8)
O1—Cu—N5—C15	150.5 (12)	N3—C11—C12—C13	−179.8 (5)
N6—Cu—N5—C15	−174.7 (5)	C11—C12—C13—C14	−4.6 (8)
O1W—Cu—N5—C15	−72.3 (5)	C11—C12—C13—N4	177.6 (5)
O7—Cu—N5—C19	−156.7 (3)	O11—N4—C13—C12	157.4 (5)
O1—Cu—N5—C19	−27.0 (15)	O12—N4—C13—C12	−21.8 (7)
N6—Cu—N5—C19	7.8 (3)	O11—N4—C13—C14	−20.5 (7)
O1W—Cu—N5—C19	110.1 (4)	O12—N4—C13—C14	160.3 (5)
O7—Cu—N6—C24	−113.5 (6)	C12—C13—C14—C9	3.3 (8)
O1—Cu—N6—C24	−7.2 (4)	N4—C13—C14—C9	−178.9 (4)
N5—Cu—N6—C24	176.9 (4)	C10—C9—C14—C13	−1.1 (7)
O1W—Cu—N6—C24	80.6 (4)	C8—C9—C14—C13	179.9 (5)
O7—Cu—N6—C20	60.4 (8)	C19—N5—C15—C16	1.3 (8)
O1—Cu—N6—C20	166.7 (3)	Cu—N5—C15—C16	−176.1 (4)
N5—Cu—N6—C20	−9.2 (3)	N5—C15—C16—C17	0.5 (9)
O1W—Cu—N6—C20	−105.5 (4)	C15—C16—C17—C18	−0.7 (9)
Cu—O1—C1—O2	8.5 (7)	C16—C17—C18—C19	−0.7 (9)
Cu—O1—C1—C2	−171.8 (3)	C15—N5—C19—C18	−2.9 (7)
O2—C1—C2—C3	−170.3 (5)	Cu—N5—C19—C18	174.8 (4)
O1—C1—C2—C3	10.0 (7)	C15—N5—C19—C20	177.1 (4)
O2—C1—C2—C7	7.8 (7)	Cu—N5—C19—C20	−5.2 (5)
O1—C1—C2—C7	−172.0 (5)	C17—C18—C19—N5	2.6 (8)
C7—C2—C3—C4	1.3 (7)	C17—C18—C19—C20	−177.4 (5)
C1—C2—C3—C4	179.3 (4)	C24—N6—C20—C21	2.3 (7)
C2—C3—C4—C5	−1.2 (7)	Cu—N6—C20—C21	−172.1 (4)
C2—C3—C4—N1	179.3 (4)	C24—N6—C20—C19	−176.6 (4)
O3—N1—C4—C5	−161.2 (5)	Cu—N6—C20—C19	9.1 (5)
O4—N1—C4—C5	17.8 (7)	N5—C19—C20—N6	−2.6 (7)
O3—N1—C4—C3	18.3 (7)	C18—C19—C20—N6	177.4 (4)
O4—N1—C4—C3	−162.7 (5)	N5—C19—C20—C21	178.6 (4)
C3—C4—C5—C6	0.4 (7)	C18—C19—C20—C21	−1.4 (8)
N1—C4—C5—C6	179.9 (4)	N6—C20—C21—C22	−1.2 (8)
C4—C5—C6—C7	0.3 (8)	C19—C20—C21—C22	177.5 (5)
C4—C5—C6—N2	178.6 (5)	C20—C21—C22—C23	−0.5 (8)
O5—N2—C6—C7	−2.9 (8)	C21—C22—C23—C24	1.1 (8)
O6—N2—C6—C7	176.9 (5)	C20—N6—C24—C23	−1.7 (7)
O5—N2—C6—C5	178.7 (5)	Cu—N6—C24—C23	172.0 (4)
O6—N2—C6—C5	−1.5 (8)	C22—C23—C24—N6	0.0 (8)
C5—C6—C7—C2	−0.2 (8)	C4S—O1S—C1S—C2S	37.4 (10)
N2—C6—C7—C2	−178.5 (5)	O1S—C1S—C2S—C3S	−34.0 (10)
C3—C2—C7—C6	−0.6 (8)	C1S—O1S—C4S—C3S	−26.8 (13)
C1—C2—C7—C6	−178.7 (5)	O1S—C4S—C3S—C2S	5.2 (14)
Cu—O7—C8—O8	−2.2 (8)	C1S—C2S—C3S—C4S	16.9 (12)
Cu—O7—C8—C9	177.9 (3)	C8S—O2S—C5S—C6S	17 (2)
O8—C8—C9—C10	1.5 (8)	C5S—O2S—C8S—C7S	4(2)
O7—C8—C9—C10	−178.6 (5)	O2S—C8S—C7S—C6S	−23 (3)
O8—C8—C9—C14	−179.6 (5)	C8S—C7S—C6S—C5S	30 (3)
O7—C8—C9—C14	0.3 (7)	O2S—C5S—C6S—C7S	−30 (3)

### Hydrogen-bond geometry (Å, °)

**Table:**

*D*—H···*A*	*D*—H	H···*A*	*D*···*A*	*D*—H···*A*
O1w—H1w···O2^i^	0.84 (5)	2.01 (6)	2.766 (6)	150 (7)
O1w—H2w···O8^i^	0.84 (5)	2.35 (7)	3.048 (6)	141 (6)
C18—H18···O8^ii^	0.95	2.17	3.060 (7)	155
C21—H21···O2^ii^	0.95	2.41	3.087 (7)	128
C15—H15···O9^i^	0.95	2.43	3.285 (7)	150
C1s—H1s2···O7	0.99	2.53	3.451 (10)	155
C3—H3···O1s	0.95	2.58	3.520 (8)	169
C2s—H2s2···O11	0.99	2.49	3.385 (11)	150
C5—H5···O4^iii^	0.95	2.58	3.360 (7)	140
C12—H12···O12^iii^	0.95	2.43	3.263 (7)	146
C16—H16···O12^iv^	0.95	2.47	3.234 (8)	138

Symmetry codes: (i) *x*, *y*, *z*−1; (ii) −*x*+1/2, *y*, *z*−1/2;  (iii) −*x*+3/2, *y*, *z*+1/2; (iv) −*x*+1, −*y*+2, *z*−1/2.

## References

[bb1] Addison, A. W., Rao, T. N., Reedijk, J., van Rijn, J. & Verschoor, G. C. (1984). *J. Chem. Soc. Dalton Trans.* pp. 1349–1356.

[bb2] Brandenburg, K. (2006). *DIAMOND* Crystal Impact GbR, Bonn, Germany.

[bb3] Bruker (2009). *APEX2* and *SAINT* Bruker AXS Inc., Madison, Wisconsin, USA.

[bb4] Farrugia, L. J. (1997). *J. Appl. Cryst.* **30**, 565.

[bb5] Flack, H. D. (1983). *Acta Cryst.* A**39**, 876–881.

[bb6] Fountain, C. S. & Hatfield, W. E. (1965). *Inorg. Chem.* **4**, 1368–1370.

[bb7] Ozair, L. N., Abdullah, N., Khaledi, H. & Tiekink, E. R. T. (2010). *Acta Cryst.* E**66**, m589–m590. 10.1107/S1600536810015060 PMC2979036 21579064

[bb8] Sheldrick, G. M. (1996). *SADABS* University of Göttingen, Germany.

[bb9] Sheldrick, G. M. (2008). *Acta Cryst.* A**64**, 112–122. 10.1107/S0108767307043930 18156677

[bb10] Westrip, S. P. (2010). *J. Appl. Cryst.* **43**, 920–925.

